# *Listeria monocytogenes* varies among strains to maintain intracellular pH homeostasis under stresses by different acids as analyzed by a high-throughput microplate-based fluorometry

**DOI:** 10.3389/fmicb.2015.00015

**Published:** 2015-01-23

**Authors:** Changyong Cheng, Yongchun Yang, Zhimei Dong, Xiaowen Wang, Chun Fang, Menghua Yang, Jing Sun, Liya Xiao, Weihuan Fang, Houhui Song

**Affiliations:** ^1^College of Animal Science and Technology, Zhejiang A&F UniversityLin'an, China; ^2^Zhejiang Provincial Key Laboratory of Preventive Veterinary Medicine, Zhejiang University Institute of Preventive Veterinary MedicineHangzhou, China

**Keywords:** *Listeria monocytogenes*, acid tolerance, intracellular pH, SigB, pH homeostasis

## Abstract

*Listeria monocytogenes*, a food-borne pathogen, has the capacity to maintain intracellular pH (pH_i_) homeostasis in acidic environments, but the underlying mechanisms remain elusive. Here, we report a simple microplate-based fluorescent method to determine pH_i_ of listerial cells that were prelabeled with the fluorescent dye carboxyfluorescein diacetate *N*-succinimidyl ester and subjected to acid stress. We found that *L. monocytogenes* responds differently among strains toward organic and inorganic acids to maintain pH_i_ homeostasis. The capacity of *L. monocytogenes* to maintain pH_i_ at extracellular pH 4.5 (pH_ex_) was compromised in the presence of acetic acid and lactic acid, but not by hydrochloric acid and citric acid. Organic acids exhibited more inhibitory effects than hydrochloric acid at certain pH conditions. Furthermore, the virulent stains *L. monocytogenes* EGDe, 850658 and 10403S was more resistant to acidic stress than the avirulent M7 which showed a defect in maintaining pH_i_ homeostasis. Deletion of *sigB*, a stress-responsive alternative sigma factor from 10403S, markedly altered intracellular pH_i_ homeostasis, and showed a significant growth and survival defect under acidic conditions. Thus, this work provides new insights into bacterial survival mechanism to acidic stresses.

## Introduction

*Listeria monocytogenes* is a Gram-positive foodborne pathogen that is responsible for severe and often life-threatening disease with high mortality (Vazquez-Boland et al., 2001; Corr and O'neill, 2009). *L monocytogenes* grows optimally in the pH ranging from 6.0 to 7.0 (Tessema et al., 2012). However, acidic environments are the common conditions encountered by listeria outside (e.g., acidic foods and soil) or inside the host (e.g., stomach and phagosomes of macrophages) (Cotter and Hill, 2003; Gray et al., 2006). This may have enabled *L. monocytogenes* to evolve a capability to grow over a wide range of pH from 4.3 to 9.4 (Te Giffel and Zwietering, 1999).

Organic acids are natural antimicrobials that have been widely used in the food industry to inhibit growth of important microbial pathogens such as *Listeria monocytogenes* and *Escherichia coli* (Carpenter and Broadbent, 2009; Otto et al., 2011). Protonated organic acids diffuse across cell membranes more freely than inorganic molecules, thus decreasing pH_i_ of the cell due to the dissociated protons (Young and Foegeding, 1993; Tessema et al., 2012). However, *L. monocytogenes* apparently adapts a resistance to acidic stress through multiple mechanisms. For example, glutamate decarboxylase (GAD), which consumes intracellular protons by converting glutamate to γ-aminobutyrate (Cotter et al., 2001a; Karatzas et al., 2012), has been suggested as an alternative acid resistance system of *L. monocytogenes* for its survival in low pH foods (Cotter et al., 2001b). Nevertheless, ammonia produced through arginine deiminase (ADI) and agmatine deiminase (AgDI) systems was found to neutralize intracellular protons by releasing NH^+^_4_ to elevate cytoplasmic pH, thereby protecting *L. monocytogenes* from lethal acidic stresses aroused from extracellular environments (Ryan et al., 2009; Chen et al., 2011a; Cheng et al., 2013a,b).

*L. monocytogenes* could maintain its intracellular pH (pH_i_) within a narrow range of 7.6–8.0 when exposed to extracellular pH (pH_ex_) beyond the range (Siegumfeldt et al., 1999; Budde and Jakobsen, 2000) by an unknown mechanism. Earlier reports showed that pH_i_ of individual bacterial cells could be measured by fluorescent ratio imaging (FRIM) using a special microscope backed up by a particular software such as Metamorph (Budde and Jakobsen, 2000; Kastbjerg et al., 2009). In FRIM, the bacterial cells were labeled with the fluorescent probe 5-(6)-carboxyfluorescein diacetate *N*-succinimidyl ester (cFDA-SE). cFDA-SE is a non-fluorescence precursor that diffuses across the cell membrane. Once inside the cell, it is hydrolyzed by the intracellular esterases and converted into a fluorescent compound which exhibits varying fluorescence intensity dependent on pH only when excited at 490 nm, but not at 435 nm. Thus, the ratio of the emitted fluorescence from two excitations at 490 nm and 435 nm (R_490/435_) reflects the pH_i_ that could be calculated (Budde and Jakobsen, 2000; Fang et al., 2006; Kastbjerg et al., 2009; Smigic et al., 2009). Pan et al. (2011) examined the pH_i_ changes of cFDA-SE labeled lactic acid bacteria cells to chitosan treatment on the cuvette-based fluorometry where no curve-fitting was performed to quantify the intracellular pH (Pan et al., 2011).

Here, we report a more effective and simple high-throughput method to determine dynamic changes of pH_i_ of different *L. monocytogenes* strains under different acidic conditions. This method was then used to examine the role of SigB in intracellular pH homeostasis upon acidic stress.

## Materials and methods

### Bacterial strains and culture conditions

*Listeria monocytogenes* lineage II (EGDe Glaser et al., 2001 and 10403S) and lineage III (M7 Chen et al., 2011b and 850658) strains were retrieved from glycerol stocks maintained at −80°C, and cultured in Brain Heart Infusion broth (BHI) (Oxoid, Hampshire, England) at 37°C. BHI broth media were adjusted with the stock solutions of hydrochloric acid (HA), acetic acid (AA), citric acid (CA), lactic acid (LA) and sodium hydroxide (NaOH) to the pH as indicated. All the pH-adjusted media were freshly made, sterilized by filtration through 0.22 μm polyethersulfone membrane filters (Millipore, Boston, USA). All chemicals were obtained from Sangon Biotech (Shanghai, China), Invitrogen (California, USA), or Sigma (St. Louis, USA) at the highest purity available.

### Fluorescent staining of *L. monocytogenes* cells

Cell labeling was performed as described previously (Budde and Jakobsen, 2000). Briefly, *L. monocytogenes* strains were grown overnight at 37°C in BHI broth at pH 7.0 with shaking, and harvested by centrifugation at 5000 × g for 3 min and re-suspended to a final OD_600 nm_ of 0.6 in sterile cold 10 mM potassium phosphate buffer (pH 7.4). The cells were stained with 10 μM 5-(6)-carboxyfluorescein diacetate *N*-succinimidyl ester (cFDA-SE, Invitrogen) and incubated at 37°C for 30 min. The cell suspension was centrifuged for 5 min at 10,000 g, resuspended in 50 mM potassium phosphate buffer (pH 6.0) containing 10 mM glucose, and energized at 30°C for 30 min. Subsequently, the cell suspension was centrifuged at 10,000 × g for 5 min and resuspended in 50 mM potassium phosphate buffer (pH 6.0) containing 10 mM glucose. The labeled bacteria were used immediately for the following pH_i_ determination.

### Intracellular pH calibration under stresses by organic and inorganic acids

In order to equilibrate the intracellular pH (pH_i_) and external pH (pH_ex_) of listerial cells, ethanol (63%, v/v) was added to the stained cells to permeabilize for 30 min at 30°C(Budde and Jakobsen, 2000). Subsequently, the bacterial cells were harvested by centrifugation at 10,000 × g for 5 min and re-suspended in BHI medium with pH ranging from 5.5 to 8.0 (in 0.5 increments), adjusted by using HA, AA, CA, and LA, respectively. Fluorescence was measured by using the microplate fluorometric reader (Biotek Synergy H1, Winooski, USA). Fluorecent ratio_490/435_ was obtained by dividing fluorescence at 490 nm by that at 435 nm. The calibration curve was plotted by polynomial fitting between Ratio_490/435_ and pH_i_ of the equilibrated cells corresponding to the pH ranging from 5.5 to 8.0, respectively. All data are reported as the mean of two independent experiments, each in triplicate wells.

### Real-time measurement of bacterial intracellular pH under stresses by organic and inorganic acids

To evaluate pH_i_ dynamics of *L. monocytogenes* strains under stresses by different acids, the labeled cells were re-suspended in BHI broth, adjusted to pH 3.5, 4.5, and 5.5 with HA, AA, CA and LA, respectively, and incubated for 60 min at 37°C. The fluorescence intensity at 490 nm and 435 nm were respectively collected every 5 min, and the corresponding pH_i_ values were determined according to the Ratio_490/435_ vs. pH_i_ calibration curves of each strain under acidic environments as described above. The data are reported as the mean of two independent experiments, each in triplicate wells.

### Growth of *L. monocytogenes* under organic and inorganic acidic conditions

*L. monocytogenes* strains were grown overnight at 37°C in BHI broth at pH 7.0 with shaking. The cultures were collected by centrifugation at 5000 × g at 4°C, washed in PBS (10 mM, pH 7.4) and adjusted to 0.6 at OD_600 nm_. The bacteria were then diluted 1:50 in fresh BHI broth (pre-adjusted to pH 4.5 or 5.5 with HA, AA, CA, and LA, respectively), pipetted into microplate wells (each strain-treatment in triplicate wells) and incubated in a microplate reader at 37°C for 14 h for automatic measurement of kinetic growth at OD_600 nm_ and 1-h interval.

### Bacterial survival in lethal acid conditions

Overnight-grown *L. monocytogenes* strains 10403S, EGDe, 850658 and M7 were harvested by centrifugation at 5000 × g for 10 min at 4°C, and then washed once in PBS (10 mM, pH 7.4). The bacterial pellets were re-suspended in BHI broth (pre-adjusted to pH 3.5 by using HA, AA, CA and LA, respectively) and incubated for 60 min at 37°C. Similar experiments were employed for 30 min survival in the synthetic human gastric fluid [8.3 g proteose peptone (Oxoid), 3.5 g D-glucose, 2.05 g NaCl, 0.6 g KH_2_PO_4_, 0.11 g CaCl_2_, 0.37 g KCl, 0.05 g bile salts (Sigma), 0.1 g lysozyme and 13.3 mg pepsin (Sigma), all L^−1^; adjusted to pH 2.5 with HCl] as described previously (Cotter et al., 2001a; Cheng et al., 2013b). The survival bacterial cells were plated onto BHI agar after appropriate dilutions. The plates were incubated at 37°C for 24 h and survival rates are reported as the mean of three independent experiments, each performed in duplicate.

### Construction of *sigB* deletion mutant

A homologous recombination strategy with SOE-PCR procedure was used for in-frame deletion of the full-length *sigB* (780 bp) from *L. monocytogenes* 10403S according to the protocol as described previously (Monk et al., 2008; Cheng et al., 2013b). The DNA fragments containing homologous arms upstream and downstream of *sigB* were obtained by PCR amplification using the SOE primers listed in Table 1. Transformants were screened as described previously (Monk et al., 2008; Cheng et al., 2013b). The resulting knockout mutant was verified by sequencing and designated as ΔsigB (Figure S1).

**Table 1:** **PCR Primers used in this study**.

**Primer name**	**Primer sequence (5′-3′)**	**Product size (bp)**
sigB-a	AT*CTGCAG*GAAATCACAGGATTGTCAG	529
sigB-b	*AACTGCCTTGTTCAT*TCTCCTCCACCT	
sigB-c	*ATGAACAAGGCAGTT*GAATCAAATAATTT	561
sigB-d	GC*GAATTC*TATCTAATATATTACGCTCGAT	

*Nucleotides introduced to create restriction sites are underlined. The complementary regions of primers are italicized*.

### Statistical analysis

All data were analyzed using the two-tailed Student's *t*-test with *P* < 0.05 as statistically significant or *P* < 0.01 as of marked statistical significance.

## Results

### cFDA-SE is a stable fluorescent indicator to measure listerial intracellular pH

**Figure 1:**
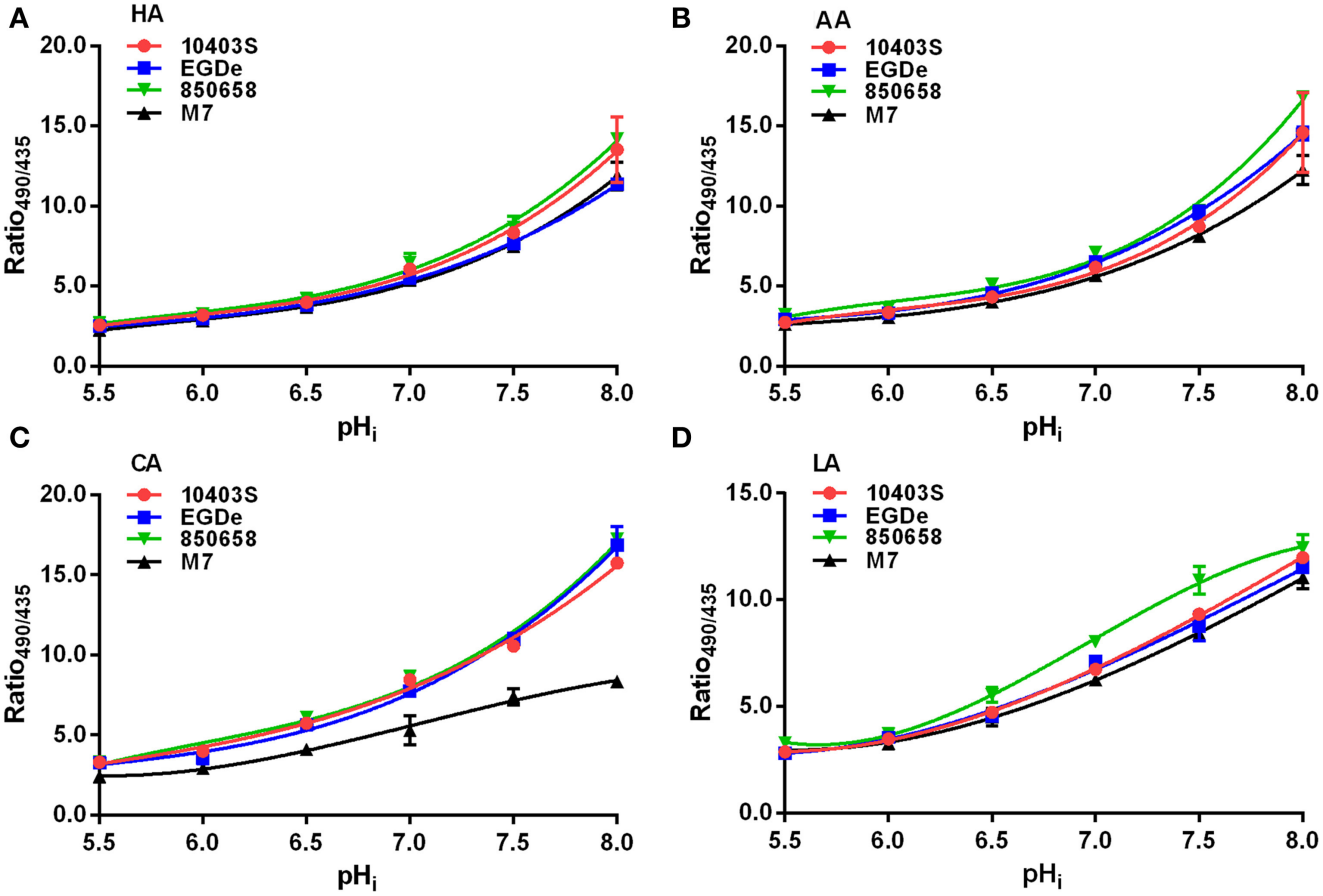
***L. monocytogenes* intracellular pH (pH_i_) determination**. *L. monocytogenes* (10403S, EGDe, 850658, and M7) strains were exposed to organic and inorganic acids HA **(A)**, AA **(B)**, CA **(C)**, and LA **(D)**. The cells were equilibrated to pH_ex_ by incubating cell preparations with ethanol and resuspending in BHI medium at certain pHs as indicated. The cells were then stained with the fluorescence dye cFDA-SE and measured in a microplate reader at 490 nm and 435 nm respectively. The pH_i_ was plotted against Ratio_490/435_. HA, Hydrochloric acid; AA, acetic acid; CA, citric acid; LA, lactic acid. Values are expressed as mean ± *SD* of two independent experiments, each in triplicate wells.

We sought to determine whether *sigB* was required for intracellular pH homeostasis of *L. monocytogenes*. To this end, it is critical to develop an accurate method to probe the intracellular pH of the bacterium. Therefore, calibration curves (Ratio_490/435_ vs. pH_i_) were plotted using ethanol-treated cells of *L. monocytogenes* under different acids (HA, AA, CA, and LA) in BHI broth for each strain (EGDe, 10403S, 850658, and M7) as indicated (Figure 1). Experimental data for each curve were found to be best fitted by a third degree polynomial equation with correlation indexes over 0.95. This indicates that the method developed in this study by using cFDA-SE as a fluorescent indicator to measure listerial pH_i_ is stable and applicable to a wide range of strains. Thus, this method was further used in the following studies to determine pH_i_ kinetics at various conditions to reveal acidic resistance of *L. monocytogenes*.

### The capability of *L. monocytogenes* to maintain intracellular pH homeostasis varies with strains, proton donors and extracellular pH

**Figure 2:**
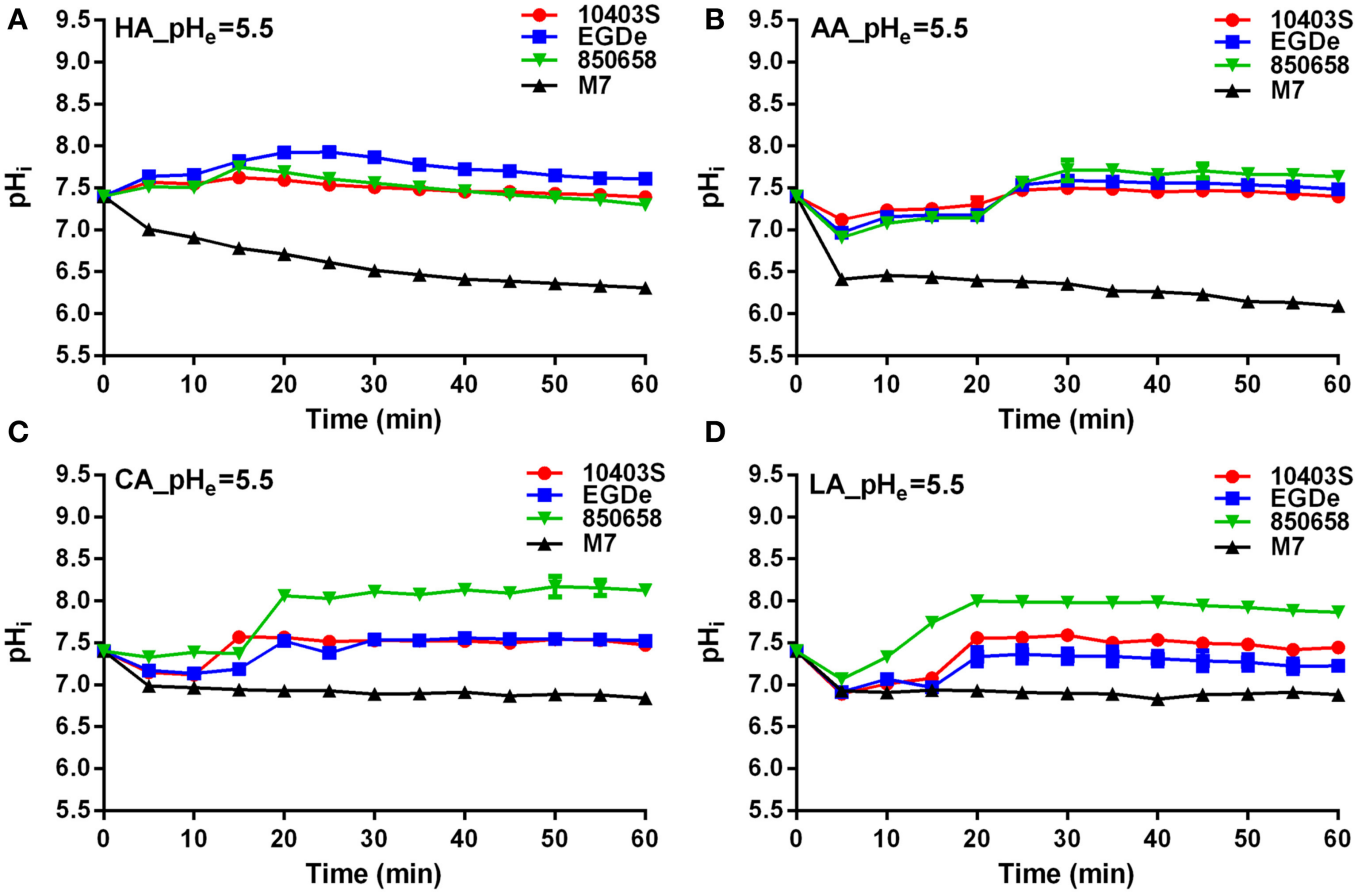
**Kinetics of intracellular pH (pH_i_) of *L. monocytogenes* strains (10403S, EGDe, 850658, and M7) exposed to organic and inorganic acids at pH 5.5**. *L. monocytogenes* strains were labeled and incubated for 60 min at 37°C in BHI broth with pH of 5.5 pre-adjusted by using HA **(A)**, AA **(B)**, CA **(C)**, and LA **(D)**, respectively. The fluorescence intensities at 490 and 435 nm were collected every 5 min, and the corresponding pH_i_ values were determined according to the Ratio_490/435_ vs. pH_i_ calibration curves (Figure 1). HA, hydrochloric acid; AA, acetic acid; CA, citric acid; LA, lactic acid. Values are expressed as mean ± *SD* of two independent experiments, each in triplicate wells.

**Figure 3:**
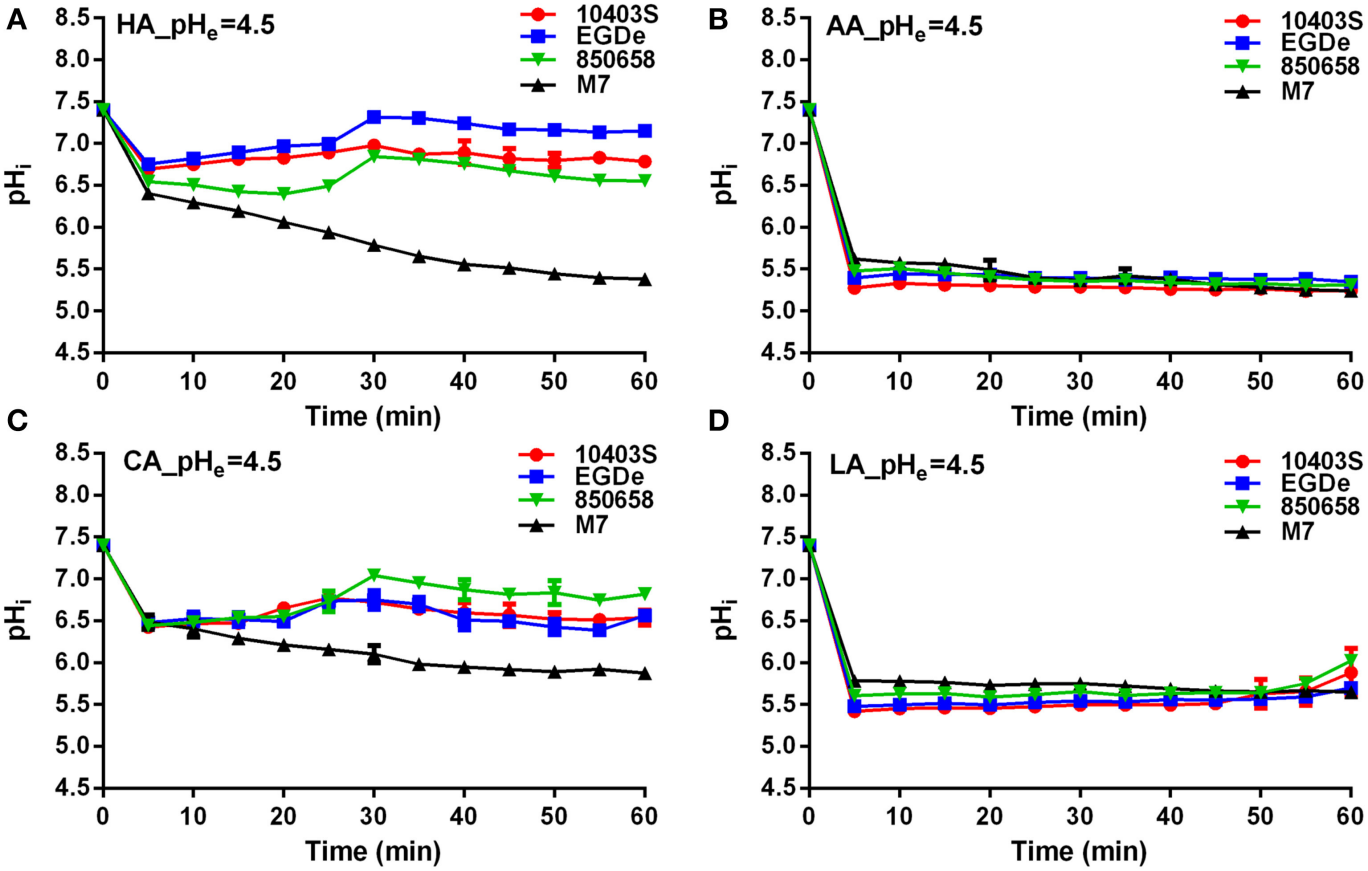
**Kinetics of intracellular pH (pH_i_) of *L. monocytogenes* strains (10403S, EGDe, 850658, and M7) exposed to organic and inorganic acids at pH 4.5**. *L. monocytogenes* strains were labeled and incubated for 60 min at 37°C in BHI broth with pH of 4.5 pre-adjusted by using HA **(A)**, AA **(B)**, CA **(C)**, and LA **(D)**, respectively. The fluorescence intensities at 490 and 435 nm were respectively collected every 5 min, and the corresponding pH_i_ values were determined according to the Ratio_490/435_ vs. pH_i_ calibration curves (Figure 1). HA, hydrochloric acid; AA, acetic acid; CA, citric acid; LA, lactic acid. Values are expressed as mean ± *SD* of three replicates.

**Figure 4:**
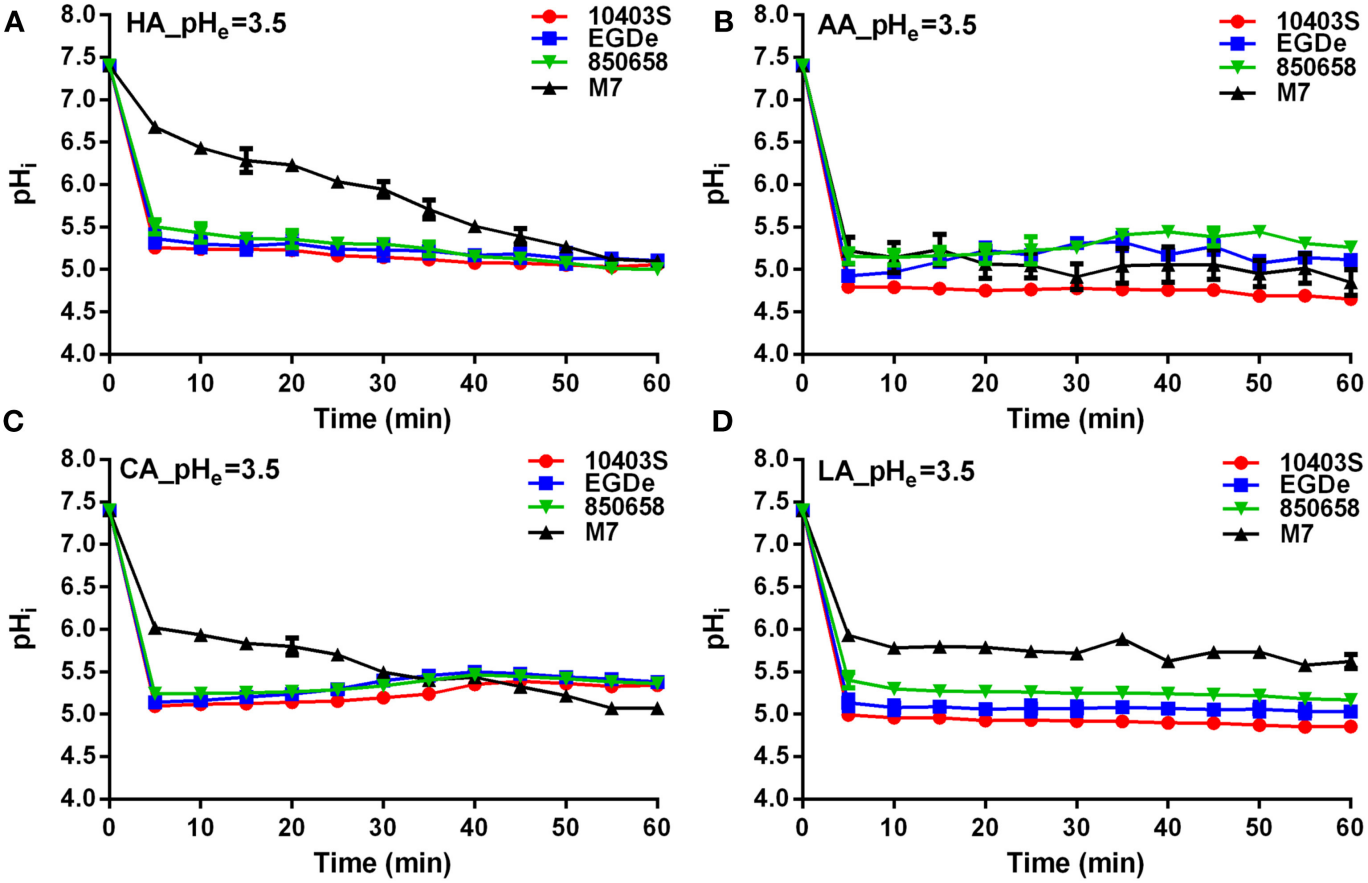
**Kinetics of intracellular pH (pH_i_) of *L. monocytogenes* strains (10403S, EGDe, 850658, and M7) exposed to the organic and inorganic acids at pH 3.5**. *L. monocytogenes* strains were labeled and incubated for 60 min at 37°C in BHI broth with pH of 3.5 pre-adjusted by using HA **(A)**, AA **(B)**, CA **(C)**, and LA **(D)**, respectively. The fluorescence intensities at 490 and 435 nm were respectively collected every 5 min, and the corresponding pH_i_ values were determined according to the Ratio_490/435_ vs. pH_i_ calibration curves (Figure 1). HA, hydrochloric acid; AA, acetic acid; CA, citric acid; LA, lactic acid. Values are expressed as mean ± *SD* of three replicates.

*L. monocytogenes* strains (virulent EGDe, 10403S, 850658, and avirulent M7) exhibited drastic variations in pH_i_ kinetics in response to different acids. Under pH 5.5 conditions, the pH_i_ of EGDe, 10403S, and 850658 strains increased rapidly after a sharp decline in the first 5 min, and maintained a steady state afterwards. However, the avirulent M7 failed to maintain its original intracellular pH when exposed to the four acids tested (Figure 2). The pH_i_ at specific time point of M7 were significantly lower than the other three strains under the same acidic conditions (Figures 2, 3). This indicates that the capability of *L. monocytogenes* to maintain intracellular pH homeostasis varied among strains at certain pH conditions. Interestingly, all listerial strains failed to maintain pH_i_ homeostasis at pH_ex_ 4.5 to the proton donor AA and LA, which was in contrast to HA and CA (Figure 3), indicating a lethal stress at this pH state induced by AA and LA. These suggest that organic and weak acids alleviate intracellular pH more effectively than inorganic and strong acids. In the case of pH 3.5, an unfavorable condition to all strains, the pH_i_ kinetics of M7 descended more slowly than other strains (Figures 4A,C,D), indicating that the M7 strain might be more resistance to HA and CA than other virulent strains at pH 3.5, although this is unlikely to happen in natural or host environments.

### Growth and survival of *L. monocytogenes* at acidic conditions varied among strains

**Figure 5:**
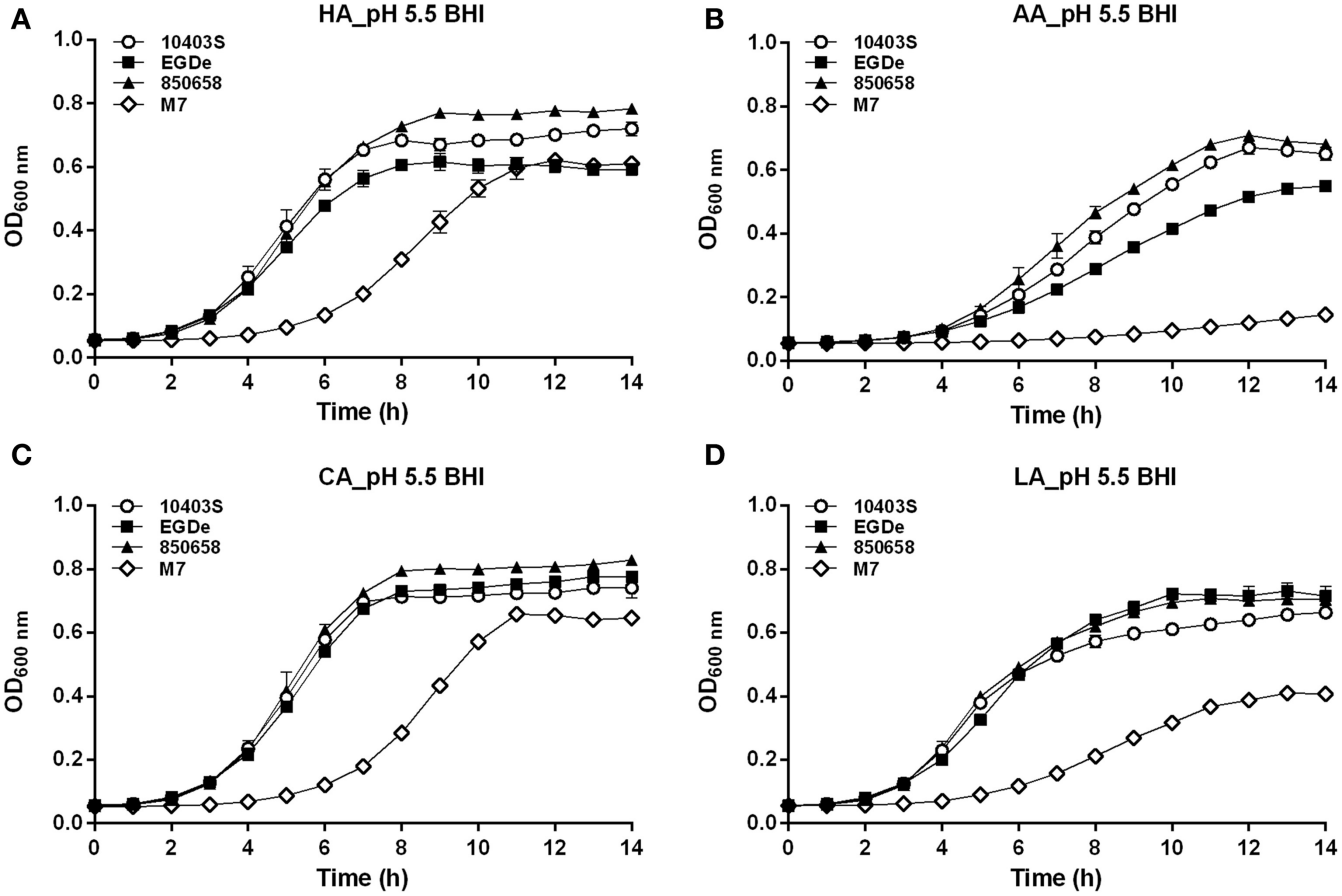
**Growth of *L. monocytogenes* strains (10403S, EGDe, 850658, and M7) exposed to organic and inorganic acids at pH 5.5**. *L. monocytogenes* strains were grown overnight at 37°C in BHI broth at pH 7.0. The cultures were collected, washed and the initial OD_600 nm_ adjusted to 0.6. The bacteria were then incubated at 37°C for 14 h in fresh BHI broth with pH of 5.5 pre-adjusted by using HA **(A)**, AA **(B)**, CA **(C)**, and LA (**D**), respectively. The kinetic growth OD_600 nm_ was then measured with 1 h interval. All experiments were performed in triplicate. HA, hydrochloric acid; AA, acetic acid; CA, citric acid; LA, lactic acid. Values are expressed as mean ± *SD* of three replicates.

**Figure 6:**
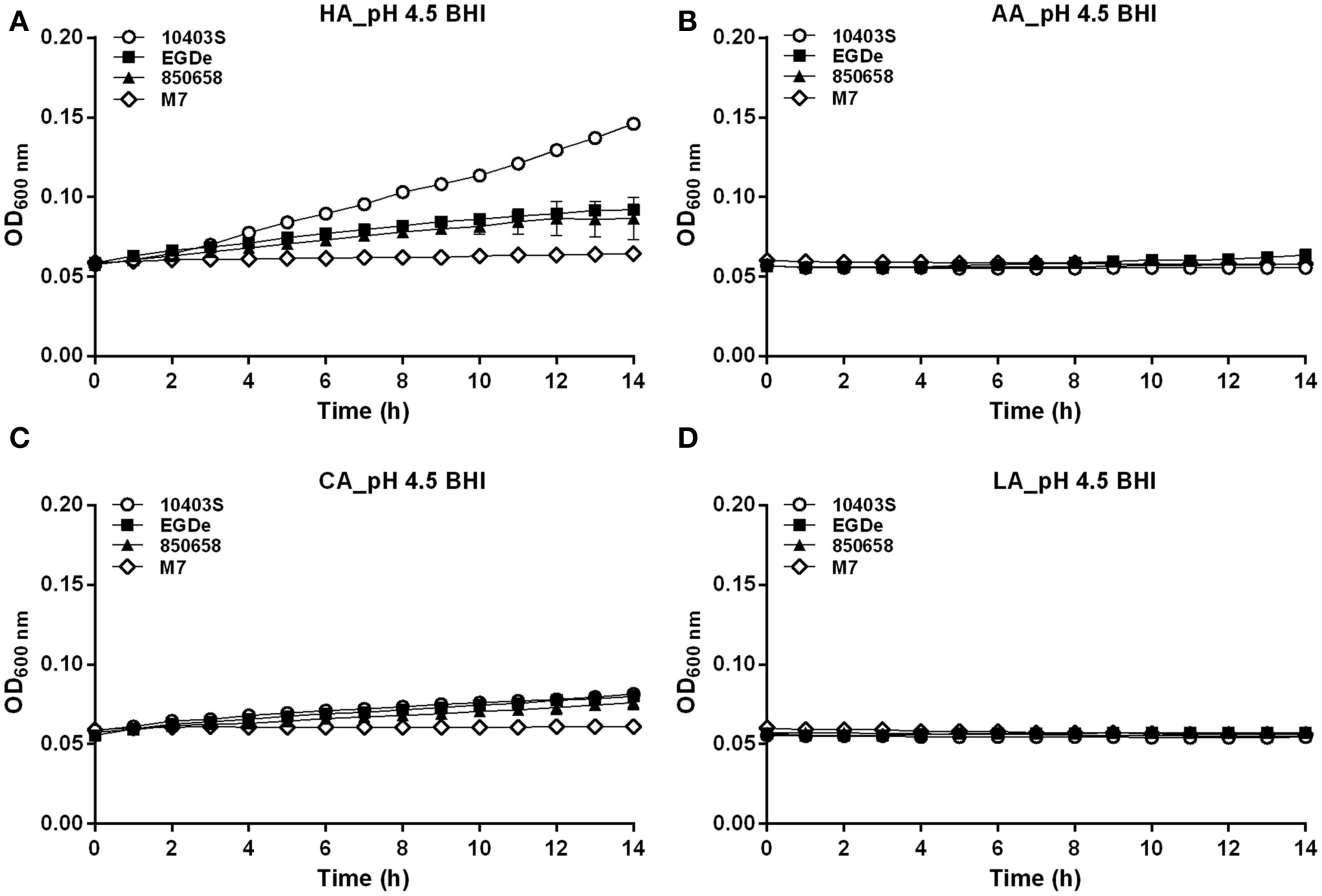
**Growth of *L. monocytogenes* strains (10403S, EGDe, 850658, and M7) exposed to organic and inorganic acids at pH 4.5**. *L. monocytogenes* strains were grown overnight at 37°C in BHI broth at pH 7.0. The cultures were collected, washed and the initial OD_600 nm_ adjusted to 0.6. The bacteria were then incubated at 37°C for 14 h in fresh BHI broth with pH of 4.5 pre-adjusted by using HA **(A)**, AA **(B)**, CA **(C)** and LA **(D)**, respectively. The OD_600 nm_ was then measured at 1 h interval. All experiments were performed in triplicate. HA, hydrochloric acid; AA, acetic acid; CA, citric acid; LA, lactic acid. Values are expressed as mean ± *SD* of three replicates.

In the BHI broth pre-adjusted to pH 5.5 by organic or inorganic acids, the growth ability of the virulent strain 10403S was nearly equal to 850658, slightly higher than EGDe (the growth order: 10403S=850658≥EGDe>>M7) (Figure 5). The avirulent strain M7 of *L. monocytogenes* showed much slower growth. In the case of pH 4.5 HA, the growth order is 10403S>EGDe=850658>>M7 (Figure 6A). Under the pH 4.5 CA, M7 almost stopped growing, but the other three strains still showed a slow yet detectable growth (Figure 6C). All strains stopped growing when exposed to AA and LA at pH 4.5 (Figures 6B,D). These results indicate that organic acids exhibited much more inhibitory effects to listerial cells than hydrochloric acid at certain pH conditions. Furthermore, M7 was more sensitive to any kind of acids compared to other four strains, which was consistent to previous pH_i_ kinetics (Figure 2).

**Figure 7:**
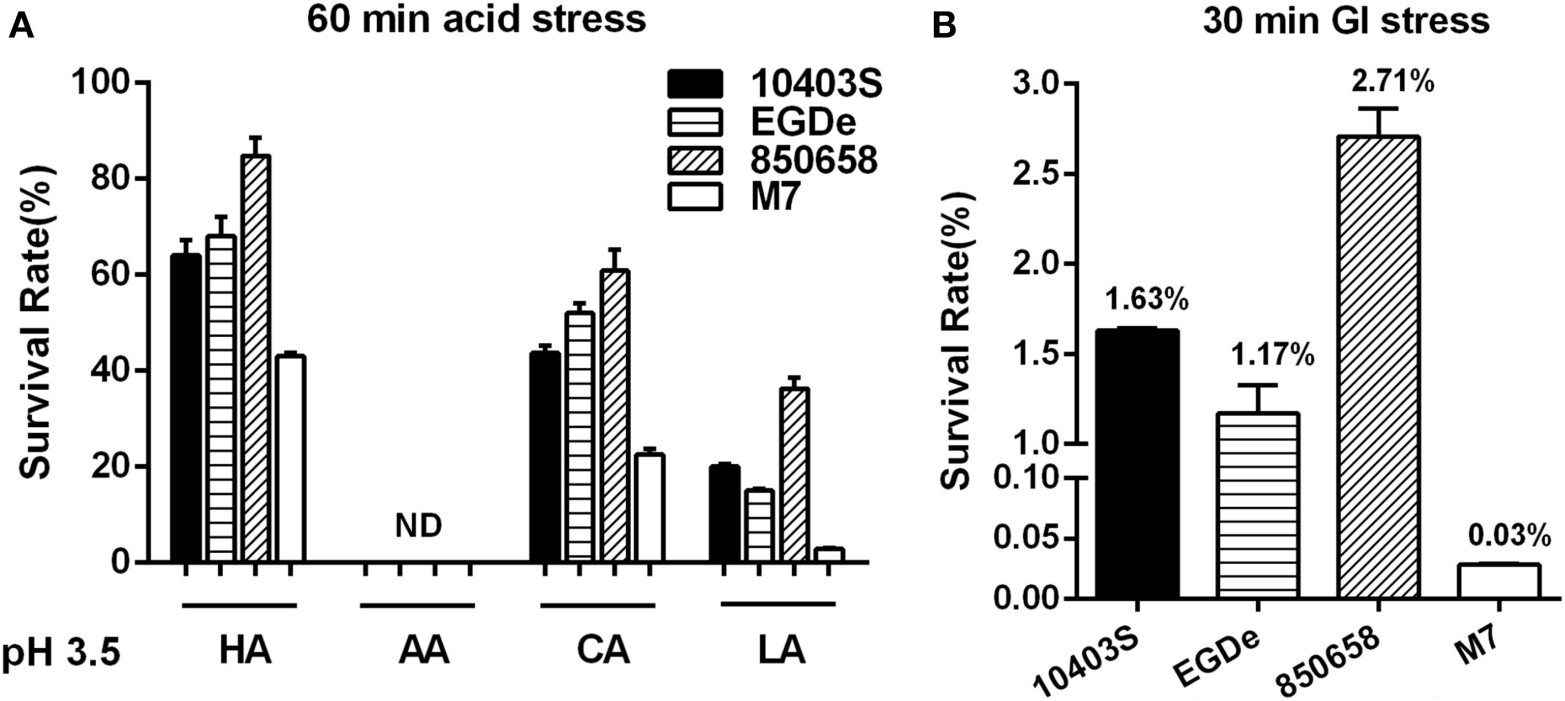
**Survival of *L. monocytogenes* strains (10403S, EGDe, 850658, and M7) in organic and inorganic acids at pH 3.5 (A) and in synthetic human gastric fluid at pH 2.5 (B)**. Overnight-grown *L. monocytogenes* strains were harvested, washed and then incubated in BHI broth (pre-adjusted to pH 3.5 by using HA, AA, CA, and LA, respectively) for 60 min and in synthetic human gastric fluid (pH 2.5) for 30 min at 37°C. Values are expressed as mean ± *SD* of three independent experiments, each performed in duplicate.

To further determine the acid tolerance of four different *L. monocytogenes* strains in the lethal acid conditions, the strain 10403S, EGDe, 850658, and M7 were exposed to HA, AA, CA, and LA at pH 3.5 and to synthetic gastric fluid at pH 2.5, respectively. The survival rate of the virulent strain 850658 at lethal acidic conditions was the highest for HA, CA, LA, and gastric fluid as compared to strains 10403S and EGDe, whereas M7 exhibited poorest survival (Figures 7A,B).

### SigB contributes to pH_i_ homeostasis of *L. moncytogenes* at acidic conditions

**Figure 8:**
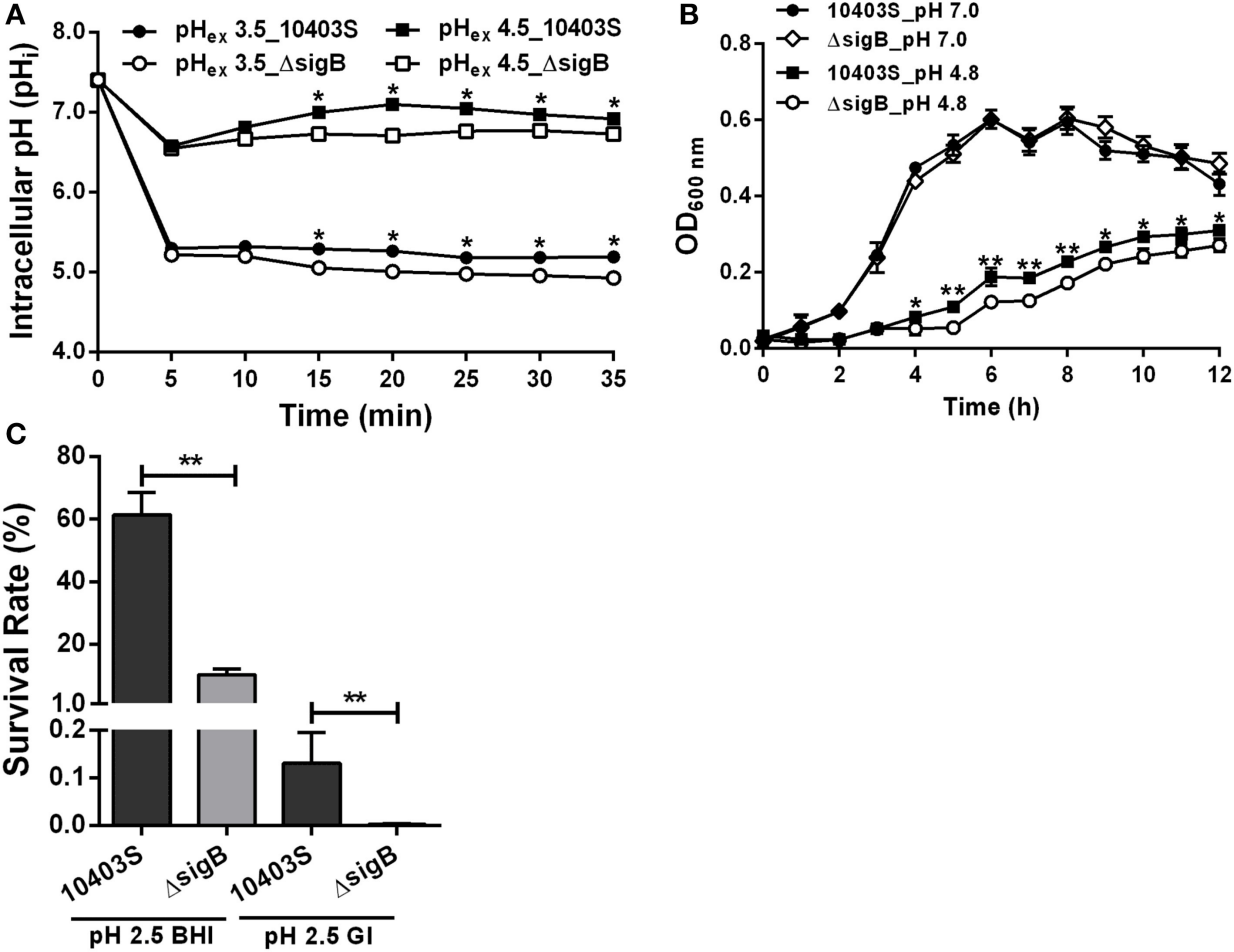
**Profiles of intracellular pH (pH_i_) (A), growth (B) and survival (C) of *L. monocytogenes sigB* deletion mutant**. HA, hydrochloric acid; AA, acetic acid; CA, citric acid; LA, lactic acid. All data are expressed as mean ± *SD* of three replicates. ^*^P < 0.05 and ^**^P < 0.01 for comparisons between the wild-type and mutant strains.

SigB was previously shown to contribute to acid tolerance response in *L. monocytogenes* (Wiedmann et al., 1998). We hypothesized that SigB is involved in maintaining *L. moncytogenes* intracellular pH. Thus, the pH_i_ dynamic of *L. moncytogenes sigB* deletion mutant was characterized by using the established method as described above. The pH_i_ of 10403S increased and then maintained stable following initial decrease in 5 min when exposed to pH_ex_ of 4.5, while *sigB* deletion mutant also showed immediate initial decline but maintained at significant lower pH_i_ than its parent strain from minutes 15 (*P* < 0.05, Figure 8A). At pH_ex_ 3.5, both the mutant and parent strains exhibited initial decline, and then maintained a lower level between 5 and 5.5 with the pH_i_ of the parent strain staying higher with statistical difference at *P* < 0.05 (Figure 8A). In addition, the growth of *L. monocytogenes* was compromised in the absence of *sigB* in the sub-lethal pH of 4.8 with a marked difference starting from hour 4 to hour 12 (*P* < 0.05), but not in the neutral pH (Figure 8B). However, deletion of *sigB* exhibited a markedly decrease in survival compared to that of its parent strain in pH 2.5 BHI or in synthetic gastric fluid (Figure 8C).

## Discussion

*L. monocytogenes* survives or even grows in a wide range of environmental conditions (Begley et al., 2010). Tolerance to low pH is important for listeria to survive because listeria encounters acidic conditions in natural and food processing environments, and in host stomach and cellular phagosome as well (O'driscoll et al., 1996). *L. monocytogenes* resists acidic stresses by up-regulating expression of specific proteins that alter cell membrane structure, increasing the bacterial ability to maintain intracellular pH (Phan-Thanh, 1998; Otto et al., 2011). SigB, a sigma factor found in Gram-positive bacteria, plays a key role in acid tolerance (Wiedmann et al., 1998; Raengpradub et al., 2008; Oliver et al., 2010; Smith et al., 2013). However, different *L. monocytogenes* strains exhibit varying abilities of acid tolerance under acidic environments, which might contribute to varying pathogenicity among strains (Conte et al., 2000; Chen et al., 2011a). This could be seen from the strain M7, an avirulent strain (Chen et al., 2011b) that was found to be more sensitive to acidic stresses than the other virulent strains in terms of growth, survival or maintenance of intracellular homeostasis.

Here, we developed a simple and high-throughput approach to measure dynamic pH_i_ changes of *L. monocytogenes* under acidic conditions by using the fluorescent dye cFDA-SE. As discussed previously, the pH range applicable to cFDA-SE dye was from 5.0 to 8.0 based on the fluorescence ratio-imaging method (FRIM) (Breeuwer et al., 1996; Budde and Jakobsen, 2000; Shabala et al., 2002; Giulitti et al., 2011). Particularly, cFDA-SE fluorescence is sensitive to pH ranging from 6.0 to 9.0. It is not sensitive enough for the FRIM to distinguish the Ratio_490/435_ between different pH gradients below 5.5 (Shabala et al., 2002). Nevertheless, the FRIM-based technique could still be extended to measure pH_i_ of 5.0 under the lethal acidic stress with pH_ex_ of 3.5 (Shabala et al., 2002; Kastbjerg et al., 2009). Therefore, we believe that cFDA-SE is applicable at pH 5.0 and can be used to measure pH_i_ even under the lethal acidic stress with pH_ex_ of 3.5, as used in this study. However, the microplate reader based method is easier and more applicable for high-throughput measurement than FRIM.

It was shown previously that protonated organic acids cross cell membrane more freely than inorganic acid molecules (Young and Foegeding, 1993; Ferreira et al., 2003). Once the disassociated protons enter inside cells, pH_i_ of the cell decreases (Bearson et al., 1997). Phan-Thanh and Montagne previously showed that when acetic acid was used to create an extracellular pH of 3.5, intracellular pH was lower than that of HCl (internal pH of 3.34 with acetic acid compared to pH of 4.22 with HCl) (Phan-Thanh, 1998). This indicates that the dissociated organic anions inside kill cells if they are not expelled or consumed. Accumulation of anions could induce cell burst if increasing osmolality and pressure persist (Carpenter and Broadbent, 2009; Otto et al., 2011). The pH_i_ of *L. monocytogenes* exposed to organic acids (acetic acid and lactic acid) is lower than that of cells exposed to HCl at the same external pH (Figures 2–4). The capacity to maintain pH_i_ homeostasis was correlated to bacterial growth and survival at acidic conditions. Therefore, we conclude that the weak acid could be used as an alternative food preservative to prevent the growth of *L. monocytogenes* and extend food shelf-life as shown previously (Le Marc et al., 2002; Lues and Theron, 2012).

Under pH 5.5, the virulent strains 10403S, EGDe and 850658 exhibited higher capacity to maintain pH_i_ homeostasis than the avirulent M7. Similar pH_i_ kinetic changes were also found at pH 4.5. Christensen and Hutkins (1992) reported that listeria cells remained viable as long as the ΔpH could be balanced. Our results showed that pH_ex_ 3.5 is close to the limit of pH_i_ homeostasis for listeria, which is consistent with the determined minimum pH_ex_ for listerial growth (Phan-Thanh et al., 2000; Le Marc et al., 2002; Shabala et al., 2006). Nevertheless, *L. monocytogenes* tends to have a buffering capacity in the cytosol around pH 5.5, which delays further pH_i_ decrease (Shabala et al., 2006). However, this buffering capacity is a short-term protection and listeria requires proton pumps to keep long-term acid tolerance (Shabala et al., 2006). Shabala et al. measured a pH_i_ of ≤ 5 after 2 h for *L. monocytogenes* incubated at pH_ex_ 3.0, and cells remained viable as these organisms recovered immediately and remained constant at pH_i_ 7.3 when returning to pH_ex_ 6.0 (Shabala et al., 2002). The ability of listeria to maintain pH_i_ homeostasis is critical for many cellular processes, such as DNA transcription, protein synthesis and enzyme activities in acidified environments (Kastbjerg et al., 2009).

SigB functions as a central regulator toward stress responses mainly through regulating expression of effector proteins (Smith et al., 2013; Ribeiro et al., 2014). When exposed to stresses, the cells respond through a regulatory cascade with the activation of σ^B^ followed by transcription of σ^B^-regulated genes involved in resistance to temperature, osmotic, chemical and pH stresses (Van Schaik and Abee, 2005; Palmer et al., 2011). However, whether SigB is involved in intracellular pH regulations is still unknown. We demonstrated that deletion of *sigB* markedly compromised intracellular pH homeostasis, and led to a significantly impaired growth and survival when the mutant strain was exposed to acidic conditions (Figure 8). Further work is still required to illustrate the mechanisms underlying the σ^B^ mediated pH_i_ homeostasis.

In summary, this study demonstrates that the microplate-based fluorometry is simple and high-throughput to measure dynamic changes of listerial pH_i_ in response to acid stresses. The method should be applicable to other bacterial species or even mutant strains involved in regulation of acid stress. We have found that *L. monocytogenes* responds differently toward organic and inorganic acids to maintain pH_i_ homeostasis. We further show that SigB plays an important role in maintaining intracellular pH homeostasis, thus providing an insight to reveal the underlying mechanisms of this central regulator in acid stress regulations in *L. monocytogenes*.

### Conflict of interest statement

The authors declare that the research was conducted in the absence of any commercial or financial relationships that could be construed as a potential conflict of interest.
