# Supplementary material for: Listeria monocytogenes varies among strains to maintain intracellular pH homeostasis under stresses by different acids as analyzed by a high-throughput microplate-based fluorometry
Source: Front Microbiol. 2015 Jan 23;6:15. doi: 10.3389/fmicb.2015.00015 (PMC4304241; doi:10.3389/fmicb.2015.00015)
Supplement: Supplementary file 1 [file Image1.PDF]

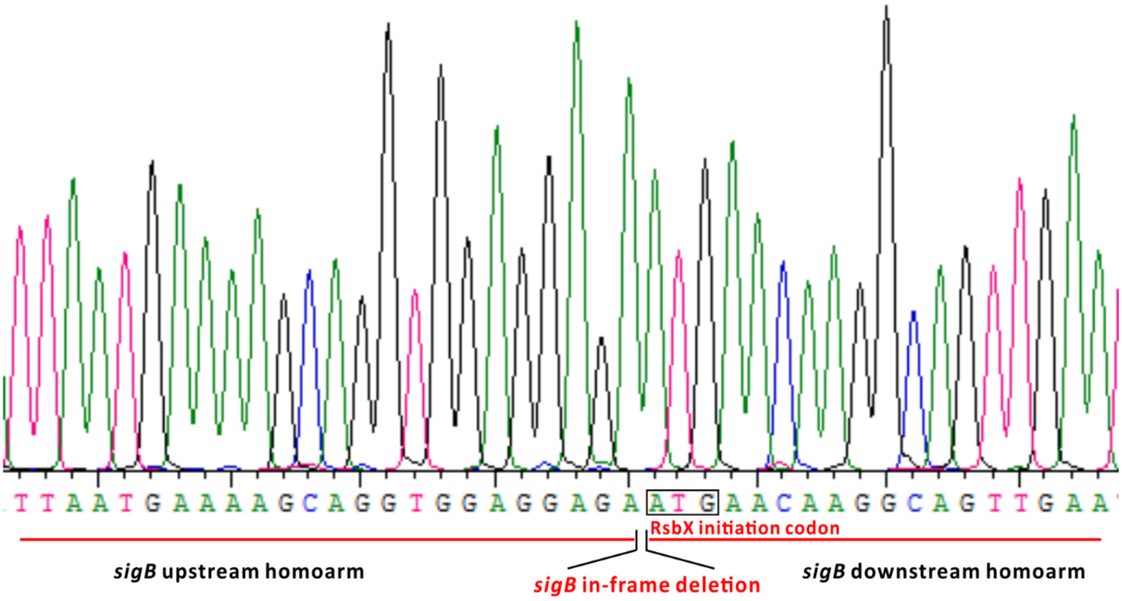

Figure S1: The sequence chromatogram of the *sigB* deletion mutant flanking the deleted *sigB* genomic fragment. The initiation codon of the cognate RsbX is indicated in a box.
